# Successful pregnancy with restorative reproductive medicine after 16 years of infertility, three recurrent miscarriages, and eight unsuccessful embryo transfers with *in vitro* fertilization/intracytoplasmic sperm injection: a case report

**DOI:** 10.1186/s13256-022-03465-w

**Published:** 2022-06-22

**Authors:** Phil C. Boyle, Joseph B. Stanford, Ivana Zecevic

**Affiliations:** 1 NeoFertility Clinic, Suite 7, 1st Floor, Beacon Mall, Sandyford, Dublin 18, Ireland; 2 Division of Public Health, Department of Family and Preventive Medicine, University of Utah School of Medicine, Salt Lake City, USA; 3 NeoFertility, Zagreb, Croatia

**Keywords:** Infertility, Polycystic ovarian syndrome, Repeated failed IVF, Recurrent miscarriage, Case report

## Abstract

**Background:**

Restorative reproductive medicine represents a comprehensive approach to subfertility (infertility and miscarriage) with investigations, diagnoses, and treatments combined with fertility charting to restore optimal reproductive function. Restorative reproductive medicine assumes that multiple factors need to be identified and treated (cycle optimization) for up to 12 cycles to achieve a successful pregnancy. Conception can occur during normal intercourse without intrauterine insemination or *in vitro* fertilization.

**Case presentation:**

A 35-year-old Croatian female presented for fertility treatment in May 2019 with a previous diagnosis of polycystic ovaries, infertility of 16 years duration, and 8 unsuccessful embryo transfers with *in vitro* fertilization and intracytoplasmic sperm injection. She was gravida 3 para 0, with 2 miscarriages after spontaneous conception at 5–6 weeks gestation in 2002 and 2004, followed by a miscarriage after *in vitro* fertilization at 12 weeks gestation in 2011. We initially found poor follicle function and suboptimal progesterone levels. Restorative reproductive medicine treatment resulted in conception after two cycles of treatment. This pregnancy ended in miscarriage at 7 weeks 4 days. Additional investigations found a balanced Robertsonian translocation (13, 14) and a uterine septum. We achieved repeat fertilization with restorative reproductive medicine after three cycles of treatment following resection of the uterine septum and ovulation induction with letrozole and human chorionic gonadotrophin. She had a full-term healthy pregnancy and live birth in 2021.

**Conclusion:**

We propose that a full evaluation of underlying factors, and up to 12 cycles of cycle optimization, should be offered to subfertile patients before considering *in vitro* fertilization treatment.

## Introduction

Involuntary childlessness affects 1 couple in every 6. It is a common problem and can result in years of expensive investigations and treatment. Many couples choose *in vitro* fertilization (IVF) to try to solve their infertility. In our practice, we use a strategy of restorative reproductive medicine (RRM) to investigate and treat subfertility [1, 2]. We ask the women/couples to learn to identify the day of presumed ovulation using a combination of cervical mucus, a rise in basal body temperature, and urinary luteinizing hormone (LH) assessment [3]. In our experience, this is best achieved with the support of a specially trained fertility advisor/teacher. Our couples typically need at least three sessions of specific instruction with additional ongoing support from their teacher. They usually record their fertility chart with our charting app at www.ChartNeo.com.

Using key information from the fertility chart, we assess the quality of ovulation initially on the basis of measuring the maximum surge of progesterone and estradiol, 7 days after presumed ovulation, and later confirmed by ultrasound. This is a significant departure from the typical day 21 progesterone blood test. Clinically, we find that day 21 progesterone testing is a crude assessment, with minimal benefit, because the actual day of ovulation can occur at or after day 21 in a substantial number of cycles [4]. In addition, simply using a threshold value for ovulation does not necessarily indicate optimized follicular function [5, 6]. Using our new approach, we ask two key questions: First, do you have optimal progesterone and estradiol 6–9 days after presumed ovulation? We use higher values and insist that progesterone must exceed 60 nmol/l (19 ng/ml) and estradiol exceed 400 pmol/l (109 pg/ml) for a healthy or optimal ovulation event. Blood levels below this range are considered suboptimal with a higher likelihood of subfertility (infertility and miscarriage). The second question we ask is: Have you had proven follicle rupture by ultrasound? This is necessary because we often see a lack of follicular rupture in subfertile patients [7]. See Table 1.

**Table 1:** Assessing the quality of ovulation

Optimal ovulation	
1. Measure both estradiol and progesterone	✔
2. Draw the serum level 6–9 days after the ovulation marker (not day 21)	✔
3. Assess optimal levels, not threshold level	✔
4. Confirm mature follicle size and follicle rupture by ultrasound in at least one cycle	✔

This case demonstrates the chronic nature of subfertility that requires multiple and sustained interventions over several cycles to restore optimal reproductive function. Ideally RRM treatment should be considered prior to IVF, but it may also be effective after years of infertility and repeated unsuccessful embryo transfers. We hope that this case may encourage doctors working with subfertility to consider additional investigations and treatments rather than progressing to IVF prematurely.

## Case presentation

### Patient information

A 35-year-old Croatian female, gravida 3 para 0, presented to our practice in March 2019 with a previous diagnosis of polycystic ovaries, 16 years of infertility, and 8 unsuccessful embryo transfers between December 2008 and December 2012. She does not smoke or drink alcohol and exercises every week. She successfully completed secondary level education and enjoys her work as an accountant and businesswoman. She lives in a small village, works indoors, and is not exposed to toxic chemicals or radiation. She was taking levothyroxine 25 mcg daily for an underactive thyroid and previously had a mole removed but otherwise no surgical or medical problems. She did not previously use hormonal contraception. She is the eldest of three sisters; one had a previous miscarriage and no live births.

### Clinical findings

Her height was 165 cm and weight 97 kg, giving a BMI of 35.6 kg/m^2^. Physical examination was otherwise unremarkable.

### Timeline

She was married at age 20 years, and after 4 months, in November 2002, she had her first pregnancy without fertility treatment. She spontaneously miscarried at 6 weeks gestation. She conceived again over 1 year later and had a second miscarriage at 5 weeks gestation in February 2004. She was diagnosed with polycystic ovary syndrome (PCOS) in 2004 and subsequently had two cycles of ovulation induction with clomiphene followed by five cycles of follicle stimulation with follicle stimulating hormone (FSH) injections without conceiving.

**Fig. 1:**
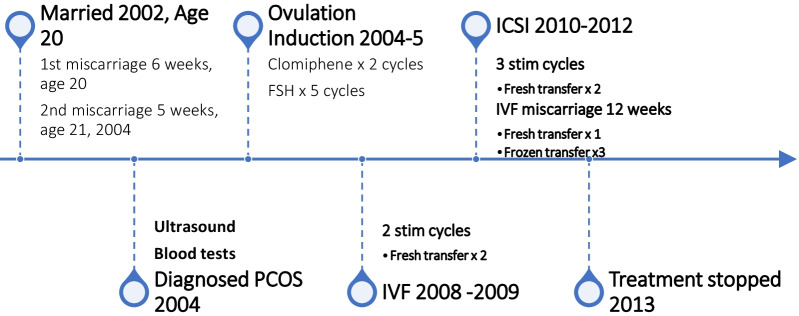
History timeline

She had two stimulated cycles of IVF in 2008 and 2009, resulting in fresh embryo transfer of two embryos each time, without success. She subsequently had three stimulated ICSI cycles until December 2012 with three additional fresh and three frozen transfers, resulting in one pregnancy that ended with miscarriage at 12 weeks gestation. In total she had five stimulated cycles with embryo transfer on eight occasions, five fresh and three frozen transfers. She stopped treatment in 2013 and resumed in 2018 (Fig. 1; Table 2).

**Table 2:** *In vitro* fertilization/intracytoplasmic sperm injection summary

Date	ICSI	IVF	Location	No. of embryos	Grade of embryos transferred (TF)	Fresh or frozen	Nonimplant	Birth, misc ectopic
1. 12/2008		x	Zagreb	2	2, 8-cells and 6-cells	Fresh	x	
2. 05/2009		x	Zagreb	2	2, stage morula	Fresh	x	
3. 07/2010	x		Zagreb	2	2, 1 morula & 8-cells	Fresh	x	
4. 11/2010	x		Zagreb	3	3, Blastocyst	Fresh		Misc.-12w
5. 05/2011			Zagreb	1	1 6-cells	Frozen	x	
6. 10/2011	x		Zagreb	2	2, 6-cells and 4-cells	Fresh	x	
7. 10/2012			Zagreb	1	1, 8-cells	Frozen	x	
8. 12/2012			Zagreb	1	1, unknown	Frozen	x	

Blood test results in March 2018, prior to attending our practice, showed anovulatory day 21 progesterone 0.49 nmol/l; day 3 blood tests were LH 7.79, FSH 6.19, TSH 3.7iu. Ultrasound revealed PCO morphology, and saline infusion sonography showed bilateral patent fallopian tubes and a normal uterus.

### Diagnostic assessment

We repeated day 3 blood tests, with the results shown in Table 3. A baseline ultrasound in her local hospital showed a normal uterus and bilateral polycystic ovaries. We confirmed polycystic ovarian syndrome based on the ultrasound, elevated DHEA-S, reversed LH to FSH ratio, and higher AMH results.

**Table 3:** Initial blood results

Blood test	Day 21	Day 3	Day 3	Normal range	
	March 18	March 18	April 19		
FSH		6.19	4.16	3.5–12.5 miu/ml	
LH		7.79	7.14	2.4–12.6 miu/ml	
TSH		3.7	2.49	0.27–4.20 miu/l	
T4			9.16	9.0–19.0 pmol/l	
Prolactin			222.7	25–629 miu/ml	
25 OH Vitamin D			53.2	30–125 nmol/l	
Haemoglobin			14.5	11.3–15.2 g/dl	
AMH			46.4	1.05–53.5 pmol/l	age 35–39 years
DHEA-S			11.04	1.6–9.25 µmol/l	
Progesterone	0.49			5.3–86.0 nmol/l	luteal phase
Oestradiol				161–774 pmol/l	luteal phase

FSH = Follicle Stimulating Hormone, LH = Luteinising Hormone, TSH = Thyroid Stimulating Hormone, T4 = Thyroxine, 25 OH Vitamin D = 25-hydroxyvitamin D, AMH = Anti Mullerian Hormone, DHEA-S = Dehydroepiandrosterone sulfate

She had 7 days of moderately severe premenstrual symptoms with irritability, breast tenderness, and bloating for 7 days before menses with relief at menstruation. She also had persistent fatigue consistent with low endorphins, which we usually treat with low-dose naltrexone [8].

**Fig. 2:**
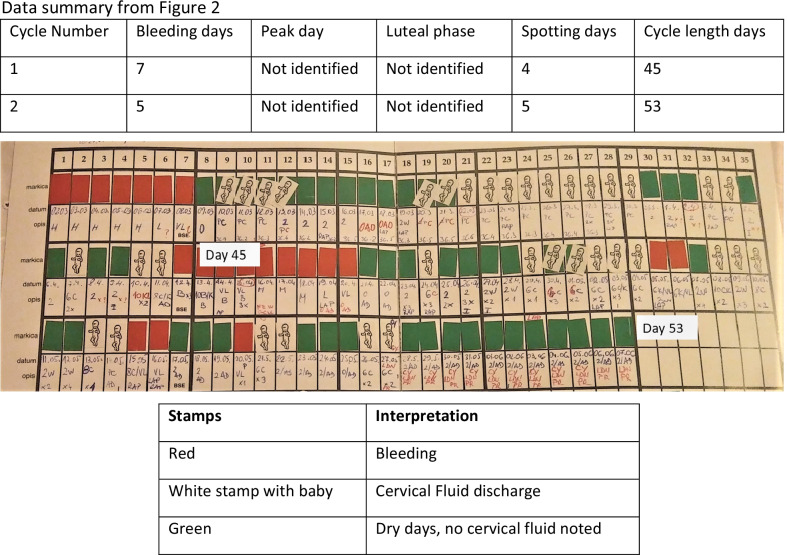
Anovulatory fertility chart, Creighton Model FertilityCare System

The patient recorded her fertility cycle and had consecutive menstrual bleeding 45 and 53 days after the first bleeding, respectively, without identifying an ovulation event. Tracking ovulation and the fertility cycle is essential to our treatment plan. Usually, we work with the NeoFertility method through our app at www.chartneo.com. In this case she began recording her cycle with the Creighton model FertilityCare System [9, 10]. See the data summary and Fig. 2

### Therapeutic interventions

We commenced treatment as follows:

1. Follicle stimulation with letrozole [11] 17.5 mg daily × 3 days from day 3 of the cycle
2. HCG 10,000 iu trigger mid cycle with 20 mm mature follicle
3. Progesterone pessaries (Cyclogest) 400 mg nightly for 10 nights from day 3 after ovulation
4. Prednisolone 5 mg [12] every morning
5. Naltrexone 4.5 mg nightly
6. Supplements with vitamin D3 4000 iu, omega 3 1000 mg, and folic acid 0.4 mg
7. Dietary strategy of low carbohydrate intake with minimal dairy and wheat
8. Metformin 500 mg twice daily with food

We opted for a higher dose of follicle stimulation in view of the patient’s longer cycles, elevated BMI, previous anovulatory day 21 progesterone levels, and no identifiable peak day from her fertility chart. For most women, we would start with letrozole 12.5–17.5 mg for just 1 day, to minimize the risk of multiple pregnancy.

**Fig. 3:**
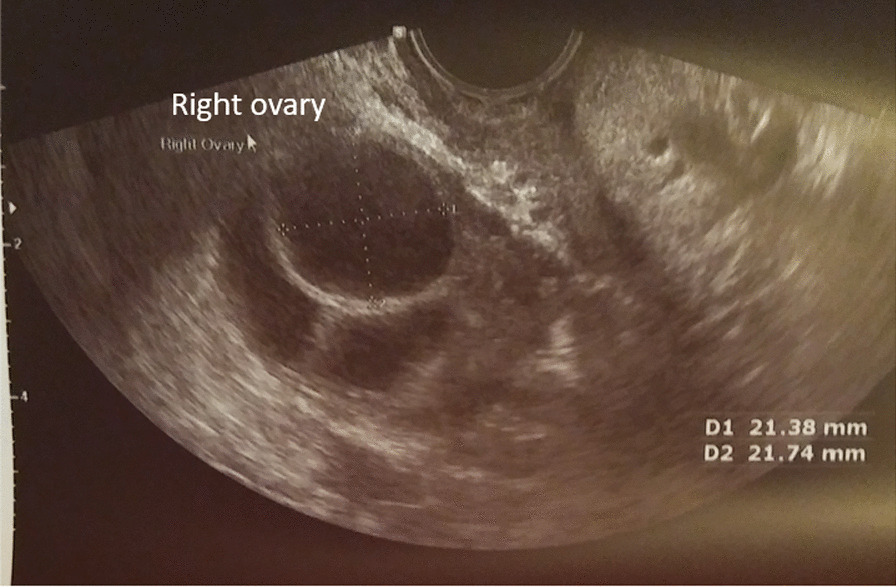
One mature follicle, with additional smaller supporting follicles

**Fig. 4:**
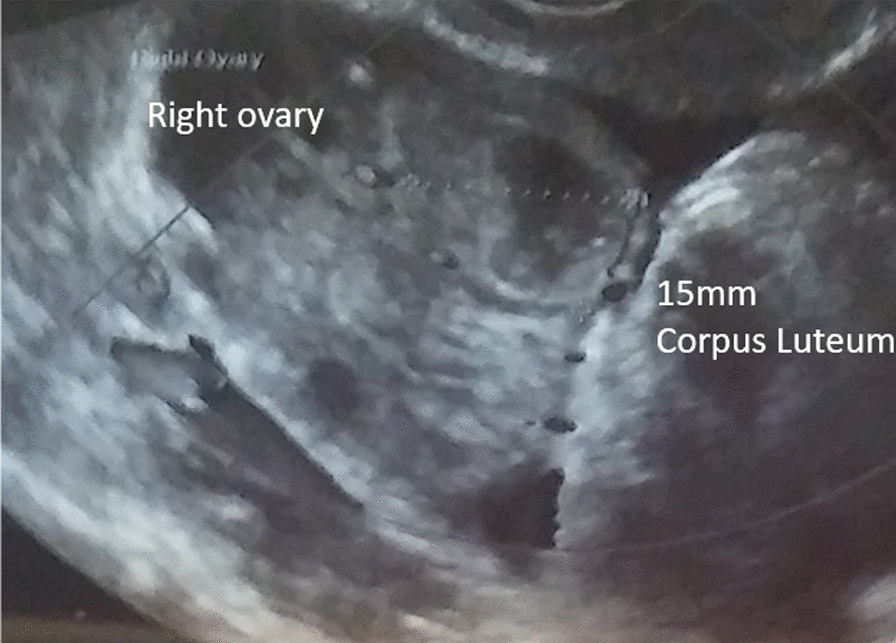
Follicle rupture

She had a confirmed mature 21 mm follicle (Fig. 3), which was proven to rupture by ultrasound follicle tracking (Fig. 4). She recruited two more follicles 14–15 mm size that did not mature or rupture.

We reduced letrozole to 12.5 mg daily × 3 days from day 3 on the second cycle of treatment, because we aimed to produce only one mature follicle each cycle.

Each day she recorded her fertility pattern with the Creighton FertilityCare system, and once a month she had a blood test for progesterone and estradiol on day 7 after ovulation, as indicated from her fertility chart.

### Follow-up outcomes

We achieved an optimal cycle with a normal-appearing fertility charting pattern, proven follicle rupture by ultrasound, and optimal levels of progesterone and estradiol on day 7 after ovulation. In addition, her fatigue and PMS symptoms improved.

We encouraged relaxed enjoyable intercourse on alternate days during the fertile time, indicated by clear slippery cervical mucus discharge.

**Fig. 5:**
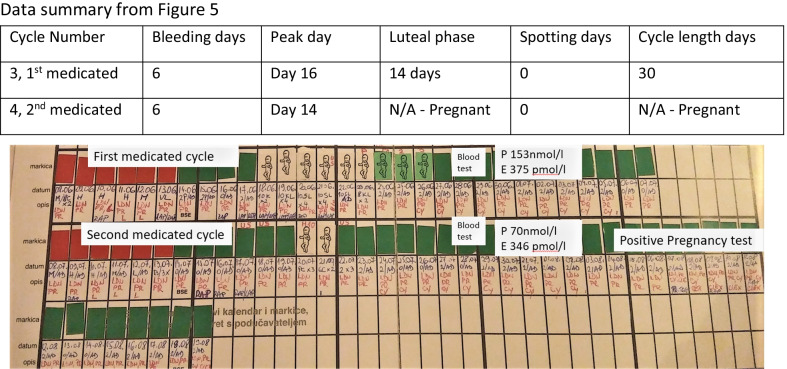
Treated cycles. Fertility observations are recorded in blue ink, while medications are recorded in red ink

They conceived on the second cycle of ovulation induction. See the data summary and Fig. 5.

Unfortunately, this pregnancy ended in miscarriage at 7 weeks 4 days when the embryonic heart stopped beating. This was managed medically with misoprostol without evacuation of retained products of conception.

Since the patient now had over three miscarriages, additional evaluation was performed over the next few months. Parental chromosome analysis found the female had a balanced Robertsonian translocation between chromosomes 13 and 14. It was estimated that her risk of future miscarriage was 25–30% each pregnancy. The patient opted for repeat treatment, knowing this risk.

She was advised to have a laparoscopy and hysteroscopy before conceiving again. Surgery was delayed due to hospital restrictions during the coronavirus disease 2019 (COVID-19) pandemic.

In June 2020, she had a hysteroscopy and was found to have a uterine septum, which was resected. This was not seen on previous ultrasound scans. A laparoscopy at the same time showed no endometriosis, bilateral patent tubes, and PCO appearance to the ovaries. She had ovarian drilling of both ovaries [13].

At the time of surgical admission her pulse was 74/minute and blood pressure 120/80. Her BMI was 34.6 kg/m^2^. Physical examination was normal. She was oriented in time, place, and situation. No focal deficits were noted. Urinalysis was clear, and blood tests for FBC, liver function, and urea and electrolytes were all normal.

We resumed medical treatment in July 2020. We discontinued metformin as she had bowel upset and tried myoinositol instead, while asking her to maintain low carbohydrate intake.

We adjusted treatment to:

1. Follicle stimulation with letrozole 12.5 mg daily × 3 days from day 3 of the cycle
2. HCG 10,000 iu trigger mid cycle with 20 mm mature follicle
3. Progesterone pessaries (Cyclogest) 400 mg nightly for 10 nights from day 3 after ovulation
4. Prednisolone 5 mg every morning and naltrexone 4.5 mg nightly
5. Vitamin D3 4000 iu, omega 3 fatty acids 1000 mg, and folic acid 0.4 mg
6. Dietary strategy of low carbohydrate intake with minimal dairy and wheat
7. Myoinositol 2000 mg twice daily 11 am and 4 pm
8. Levothyroxine 25 mcg daily

See Table 4 for a list of RRM medications used.

**Table 4:** List of RRM medications used

RRM medication	Purpose
Letrozole 2.5 mg	Follicle stimulation to improve follicle function. Titrate dose from 12.5 mg to 17.5 mg for 1–3 days, starting on day 3 of cycle. Aim to produce one follicle
HCG 10,000 iu	Bolus HCG mid cycle timed with positive LH surge, helps follicle to rupture
Cyclogest 400 mg	Taken vaginally for 10 nights from day 3 after ovulation to improve progesterone levels in the luteal phase of the cycle
Prednisolone 5 mg	Combined with letrozole, to improve follicle function in resistant cases
Metformin 500 mg	2–3 times daily, to treat insulin resistance and improve follicle function
Myoinositol 2000 mg	2 times daily, to treat insulin resistance and improve follicle function
Levothyroxine 25–75 mcg	To treat borderline underactive thyroid. Aim to keep TSH 1–2 IU
Naltrexone	To treat clinical low endorphins presenting with PMS, fatigue, and low mood

**Fig. 6:**
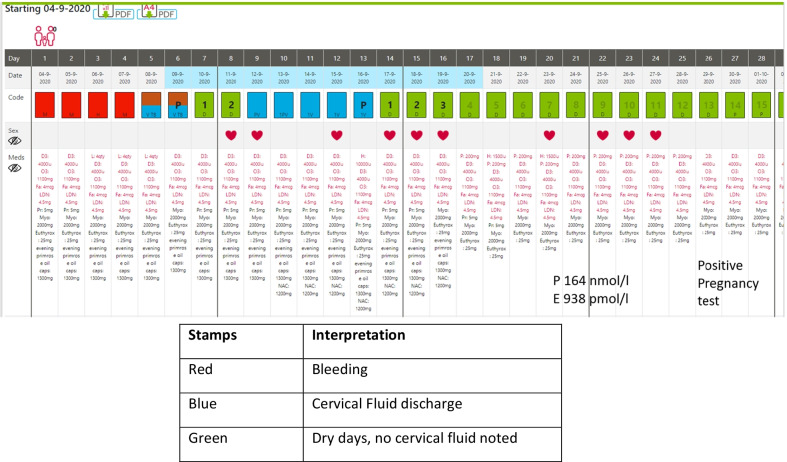
NeoFertility method, cycle of conception

She began recording her cycle with the NeoFertility method at www.chartneo.com from July 2020 (Fig. 6).

She had a repeat positive pregnancy test in September 2020, her third medicated cycle after surgery, 1 year after her first miscarriage with RRM treatment. Her body weight reduced by 7 kg to 90 kg, giving a modest reduction in BMI to 33.1 kg/m^2^. On the cycle of conception, she had treatment as follows:

1. Follicle stimulation with letrozole 10 mg daily × 3 days from day 3 of the cycle
2. HCG 10,000 iu trigger mid cycle with 20 mm mature follicle
3. Progesterone pessaries (Cyclogest) 400 mg nightly for 10 nights from day 3 after ovulation
4. Prednisolone 5 mg every morning and naltrexone 4.5 mg nightly
5. Supplements with vitamin D3 4000 iu, omega 3 fatty acids 1000 mg, and folic acid 0.4 mg
6. Dietary strategy of low carbohydrate intake with minimal dairy and wheat
7. Myoinositol 2000 mg twice daily 11 am and 4 pm
8. Levothyroxine 25 mcg daily

**Fig. 7:**
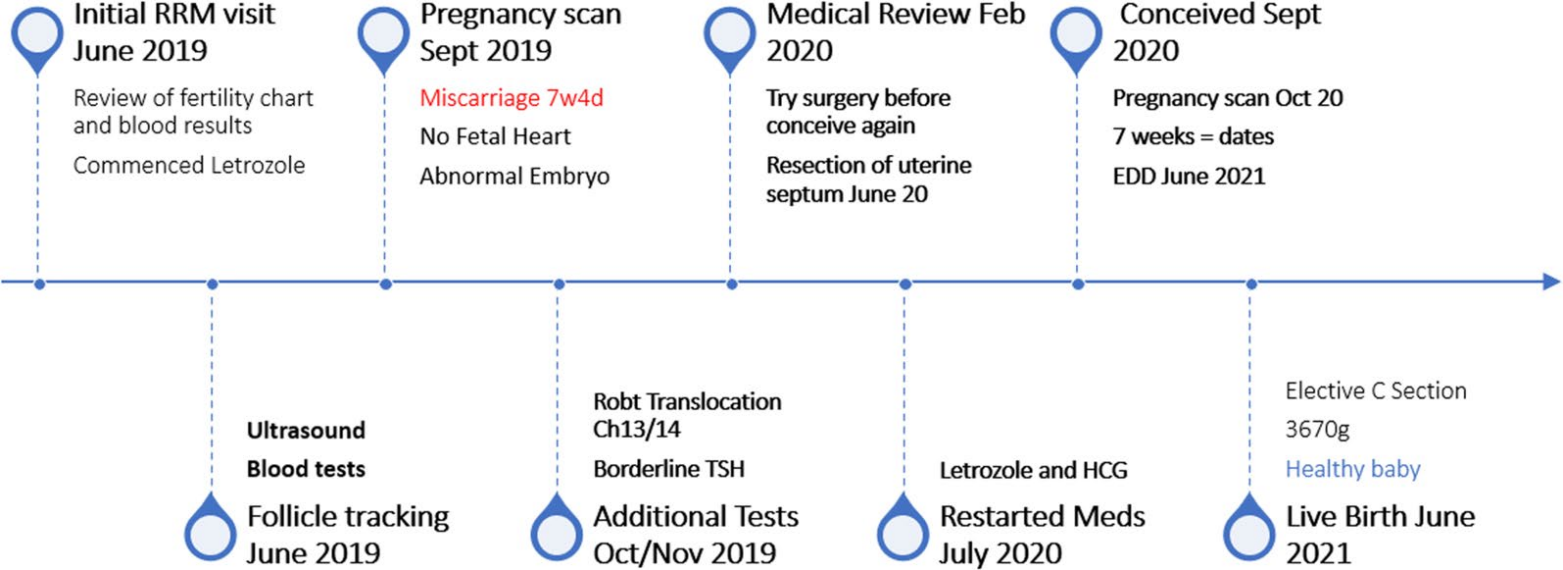
RRM treatment timeline

See Fig. 7 for a summary of RRM treatment timeline.

We increased levothyroxine to 75 mcg during pregnancy to keep TSH between 1 and 2 iu.

Ultrasound scan at 7 weeks gestation in October 2020 confirmed a viable singleton pregnancy consistent with dates and estimated date of delivery of June 2021.

She had a full-term pregnancy at 39 weeks, delivered by elective caesarean section for fetal malpresentation. She delivered a male infant, birth weight 3.670 kg. Both mother and baby were healthy.

## Discussion

Recurrent implantation failure (RIF) refers to unsuccessful implantation after repeated transfers of morphologically good-quality embryos into a normal uterus [14]. Most treatment strategies focus on refining IVF treatment with (a) better embryo selection (through preimplantation genetic screening (PGS) or time-lapse imaging during embryonic incubation), (b) enhancing endometrial receptivity with an endometrial scratch, or (c) novel immunological treatments. We propose a different treatment strategy with RRM, trying to identify and treat the root causes of subfertility and restore optimal reproductive function. A key strategy of RRM is to record the menstrual cycle with a fertility chart. In this way, we have a simple and reproducible way to identify the fertile window, bleeding pattern, day of presumed ovulation, and length of luteal phase. We also have a record of days used for intercourse to allow natural conception. Our fertility app at www.chartneo.com allows us to additionally record all medications taken and blood results each cycle. It is also useful to confirm the date of conception. The ongoing guidance and support of a trained fertility advisor/teacher is central to our success.

This case demonstrates the principles of restorative reproductive medicine (RRM).

First, with ongoing evaluation, she had multiple diagnoses requiring attention:

1. Polycystic ovarian syndrome
2. Poor follicle function
3. Low endorphins (presumptive)
4. Hypothyroidism
5. Uterine septum
6. Balanced Robertsonian translocation (Ch 13–14)

Having implemented a strategy to restore optimal reproductive function, we sought to optimize conditions for natural conception and healthy pregnancy in a comprehensive, iterative fashion, as follows:

1. Adequate follicle stimulation and rupture was verified by ultrasound, using letrozole and HCG trigger;
2. Blood tests results 7 days after ovulation were within our target range consistent with optimal ovulation;
3. Fatigue and premenstrual symptoms were greatly improved, treated with low-dose naltrexone;
4. Adequate treatment of borderline hypothyroidism;
5. Identification of previously unrecognized uterine septum, with surgical correction;
6. PCOS was addressed with low carbohydrate diet, metformin or inositol, and ovarian drilling;
7. The fertility charting pattern—bleeding, mucus, luteal phase of cycle—was normalized;
8. Other adjunctive treatments to normalize ovulation and cycle function included prednisolone, postovulatory progesterone, vitamin D, omega 3, and folic acid.

Initially, she conceived on her second optimized cycle, but miscarried. This prompted additional evaluation and intervention. We wanted surgical assessment before conceiving again, and this delayed us by 6 months due to COVID restrictions. After surgery, she had three medicated optimal cycles, conceived again, and had a successful outcome.

We identified and treated the principal factors preventing conception, and we restored normal function. This allowed the couple to conceive naturally with a lower risk of miscarriage.

A limitation with our approach is that we cannot select the best embryo free of a balanced translocation as you could with IVF and preimplantation genetic selection (PGS). It is likely our first miscarriage was due to this factor, but the second conception was healthy, resulting in a successful pregnancy.

When we achieve optimal medical and surgical conditions for pregnancy to occur, we remind couples that it is normal to take up to 12 cycles to conceive [15]. We need to have a single mature follicle that ruptures, with optimal progesterone and estradiol in the mid-luteal phase. It is important to record the fertility cycle pattern daily, check monthly blood tests for progesterone and estradiol, 7 days post ovulation, and periodically assess follicle rupture by ultrasound. We may persist for up to 12 optimized cycles, because in healthy couples with normal reproductive function, it is normal to take 12 cycles to conceive.

There has been a theoretical concern that prolonged treatment with clomiphene may increase the lifetime risk of developing ovarian cancer, through excessive stimulation of follicles [16]. To the extent that this is an important concern, we believe it would be related to the intensity of follicular stimulation. If we restore optimal cycles with one mature follicle each month, it is likely to be less harmful than ovarian stimulation for a cycle of IVF.

## Conclusion

RRM treatment seeks to produce one mature follicle and maintain optimal cycles, avoiding excessive stimulation. Many clinics discontinue ovulation induction too quickly because of concerns regarding excessive follicle stimulation. However, moving prematurely to IVF can cause more intense follicle stimulation, with a risk of ovarian hyperstimulation syndrome. In contrast, repeat optimized cycles with less stimulation are low risk and can achieve a successful pregnancy even with long-standing infertility, recurrent miscarriage, and multiple unsuccessful embryo transfers. We propose that a full evaluation of underlying factors, and up to 12 cycles of cycle optimization, should be offered to subfertile patients before considering IVF treatment.

## Data Availability

Actual blood test results and copies of the fertility charts are available for review and assessment.
